# Global scientific trends on the islet transplantation in the 21st century: A bibliometric and visualized analysis

**DOI:** 10.1097/MD.0000000000037945

**Published:** 2024-04-26

**Authors:** Sheng Chen, PeiZhong Wu, Ting Zhang, Jianqiang Zhang, Hongjun Gao

**Affiliations:** a Graduate School, Guangxi University of Chinese Medicine, Nanning, China; b Ruikang Hospital, Guangxi University of Chinese Medicine, Nanning, China.

**Keywords:** bibliometrics, CiteSpace, islet transplantation, mesenchymal stem cell, VOSviewer

## Abstract

**Background:**

Islet transplantation (IT) has emerged as a significant research area for the treatment of diabetes mellitus and has witnessed a surge in scholarly attention. Despite its growing importance, there is a lack of bibliometric analyses that encapsulate the evolution and scientific underpinnings of this field. This study aims to fill this gap by conducting a comprehensive bibliometric analysis to delineate current research hotspots and forecast future trajectories within the IT domain with a particular focus on evidence-based medicine practices.

**Methods:**

This analysis scrutinized literature from January 1, 2000, to October 1, 2023, using the Web of Science Core Collection (WoSCC). Employing bibliometric tools such as VOSviewer, CiteSpace, and the R package “bibliometrix,” we systematically evaluated the literature to uncover scientific trends and collaboration networks in IT research.

**Results:**

The analysis revealed 8388 publications from 82 countries, predominantly the United States and China. However, global cross-institutional collaboration in IT research requires further strengthening. The number of IT-related publications has increased annually. Leading research institutions in this field include Harvard University, the University of Alberta, the University of Miami, and the University of Minnesota. “Transplantation” emerges as the most frequently cited journal in this area. Shapiro and Ricordi were the most prolific authors, with 126 and 121 publications, respectively. Shapiro also led to co-citations, totaling 4808. Key research focuses on IT sites and procedures as well as novel therapies in IT. Emerging research hotspots are identified by terms like “xenotransplantation,” “apoptosis,” “stem cells,” “immunosuppression,” and “microencapsulation.”

**Conclusions:**

The findings underscore a mounting anticipation for future IT research, which is expected to delve deeper into evidence-based methodologies for IT sites, procedures, and novel therapeutic interventions. This shift toward evidence-based medicine underscores the field’s commitment to enhancing the efficacy and safety of IT for diabetes treatment, signaling a promising direction for future investigations aimed at optimizing patient outcomes.

## 1. Introduction

Hyperglycemia, the hallmark of diabetes, results from a diminished ability of the body to produce or respond to insulin, making it a complex metabolic disorder. The World Health Organization reports that 451 million people globally are currently living with diabetes, a number projected to increase to 693 million by 2045.^[1]^ The escalating burden of diabetes affects every country and its population worldwide. Consequently, extensive research has been conducted on management, control, and treatment strategies for diabetes. Currently, daily insulin injections remain the standard treatment for individuals with Type 1 Diabetes (T1D), late-stage Type 2 Diabetes, and certain rare forms of the disease.^[2]^ Although life-saving, daily insulin injections do not entirely mimic the natural blood glucose regulation of β cells. Additionally, this therapy is costly and burdensome for patients with diabetes and does not entirely eliminate the risk of acute and long-term diabetes complications. The current treatment for insulin-dependent diabetes involves pancreatic IT, which involves isolating islets from donors and infusing them percutaneously into the liver via the portal vein.^[3–8]^ Among the available therapies, IT may be the most promising approach for the clinical treatment of diabetes, particularly TD1 mellitus.^[9,10]^ In recent decades, the number of publications on IT has significantly increased, yet no bibliometric study has been conducted. Therefore, this study aims to provide a comprehensive overview of the IT field’s knowledge structure and research trends using bibliometric analysis.

Bibliometric analysis, which employs diverse statistical methods, serves as a quantitative tool for scrutinizing and appraising knowledge carriers within a specific field. It facilitates the formulation of therapeutic guidelines and aids in understanding research trends, knowledge frameworks, collaborative patterns, and prospective development directions for a given subject.^[11,12]^ This approach is widely used in many disciplines, and in this study, it seeks to summarize the features of IT literature, explore research findings and future directions for this topic, and offer standards for future investigations.^[13]^

## 2. Materials and methods

### 2.1. Database

The Web of Science Core Collection (WoSCC) is acknowledged as a premier and extensive database in scientific research, encompassing a vast array of scholarly literature and studies.^[14]^ In this study, we used WoSCC to conduct an exhaustive literature search.

### 2.2. Search strategies

On October 10, 2023, we retrieved and downloaded all papers from the WoSCC database published between 2000 and 2023 to control for daily variations in database content. Our search employed the following terms: TOPICS = (Islands of Langerhans Transplantation) OR (Transplantation, Islands of Langerhans) OR (Islands of Pancreas Transplantation) OR (Transplantation, Islands of Pancreas) OR (Transplantation, Islet) OR (Pancreatic Islets Transplantation) OR (Islets Transplantation, Pancreatic) OR (Transplantation, Pancreatic Islets) OR (Islet transplantation) OR (Islet transplantations) OR (Transplantations, Islet) OR (Grafting, Islets of Langerhans) OR (Transplantation, Islets of Langerhans).

### 2.3. Exclusion and inclusion criteria of publications

**Figure 1.**
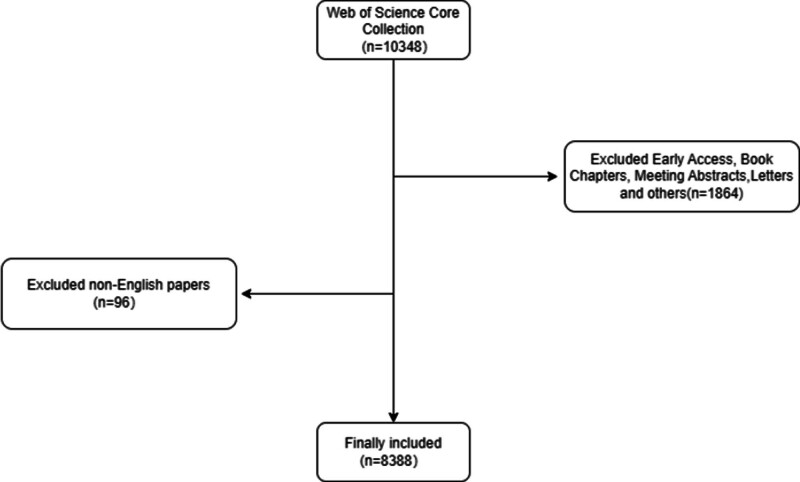
Flow diagram of the included papers.

A total of 10,348 articles on IT were retrieved from the WoSCC database. We excluded 1864 articles classified as conference abstracts, proceedings, case reports, letters, or off-topic papers. Additionally, 96 non-English articles were excluded. The final selection for this study included original studies, reviews, and meta-analyses pertinent to IT-related literature. The literature exclusion and inclusion process is shown in Figure 1. Ethical approval was not necessary for this study because it was bibliometric and did not include human or animal subjects.

### 2.4. Data extraction and analysis

To remove duplicates, plain text files from WoSCC were imported into CiteSpace. The unique files were then compiled and transferred to Microsoft Excel to analyze the authors, countries, journals, and institutions associated with the publications.

CiteSpace (version 6.1.R1), developed by Professor Chen C, is a software designed for bibliometric analysis and visualization^[15]^. In this study, we set a time frame from January 2000 to October 2023, and utilized a single time slice in CiteSpace for keyword clustering, burst word analysis, and burst reference analysis.

VOSviewer (version 1.6.18) is a tool designed for bibliometric analysis, adept at distilling essential information from numerous publications^.[16]^ In our study, VOSviewer was instrumental in conducting co-occurrence analysis across 3 modules: density, overlay, and network visualization: for institutions, authors, countries, and keywords. This facilitated the creation of a visual atlas that illustrated the connections between research hotspots and scientific literature across various dimensions. Within the VOSviewer map, each node represents an entity (such as a nation, organization, publication, or author), with the node size and color reflecting the quantity and type of these entities, respectively. In addition, the thickness of the lines between nodes indicates the degree of relatedness or co-citation of the entities.^[17,18]^

Utilizing the “bibliometrix” R package (version 3.2.1), we generated a global distribution network of topic-related articles.^[19]^

GraphPad Prism (8.0.2) was used to tally the number of issued documents and to generate line graphs.

Given the nature of this bibliometric analysis, which exclusively examined publicly available literature and did not involve direct research on humans or animals, ethical approval, and patient consent were not required for this study. This approach aligns with the guidelines and exemptions for studies requiring ethical oversight, as our work did not entail any intervention, interaction with human subjects, or the collection of personal data. Therefore, the necessity for approval from an ethics committee or institutional review board was deemed not required.

## 3. Results

### 3.1. Annual publications

**Figure 2.**
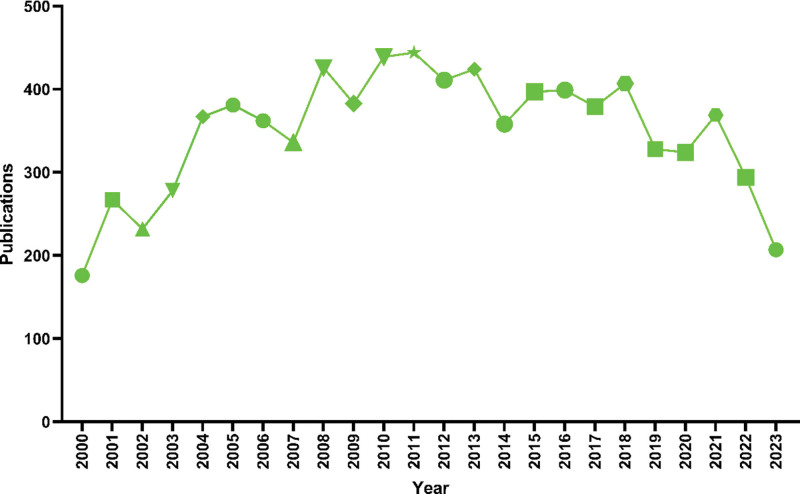
Annual publication volume trend from 2000 to 2023.

This study included a total of 8388 articles. A line graph illustrating the annual publication count is presented in Figure 2. The annual rate of publications in IT research exhibited a fluctuating upward trend, marked by significant peaks in 2004, 2011, and 2018, indicative of heightened research activity in those years. The decline in publications in 2023 can be attributed to the timing of the search, but the overall trend continues to show an upward trajectory. Notably, over the past 5 years, IT has maintained a consistent annual publication output, underscoring its status as a current research hotspot that continues to garner attention.

### 3.2. Analysis of leading journals

A total of 1155 academic journals have published papers related to IT. Table 1 displays the top 10 journals along with the number of documents they have published. Leading the list with 608 articles is “Transplantation,” followed by “Cell Transplantation” with 504 articles, “Transplantation Proceedings” with 482 articles, and the “American Journal of Transplantation” with 331 articles. Among these top 10 journals, “Diabetes” has received the highest number of references, totaling 23,402 citations, followed by “Transplantation” with 21,444 citations, “American Journal of Transplantation” with 12,849 citations, and “Cell Transplantation” with 11,392 citations. These figures underscore their significant scholarly impact in the field of information technology.

**Table 1:** The top 10 productive and cited journals on the research of IT.

Rank	Journal	Publications	Count of citations	IF (2022)
1	Transplantation	608	21,444	6.20
2	Cell Transplantation	504	11,392	3.30
3	Transplantation Proceedings	482	5271	0.90
4	American Journal of Transplantation	331	12,849	8.80
5	Diabetes	276	23,402	7.70
6	Xenotransplantation	268	5368	3.90
7	Diabetologia	191	9473	8.20
8	Plos One	174	4848	3.70
9	Biomaterials	138	9278	14.00
10	Islets	113	1397	2.20

IT = islet transplantation.

### 3.3. Analysis of leading countries, regions, and institutions

**Figure 3.**
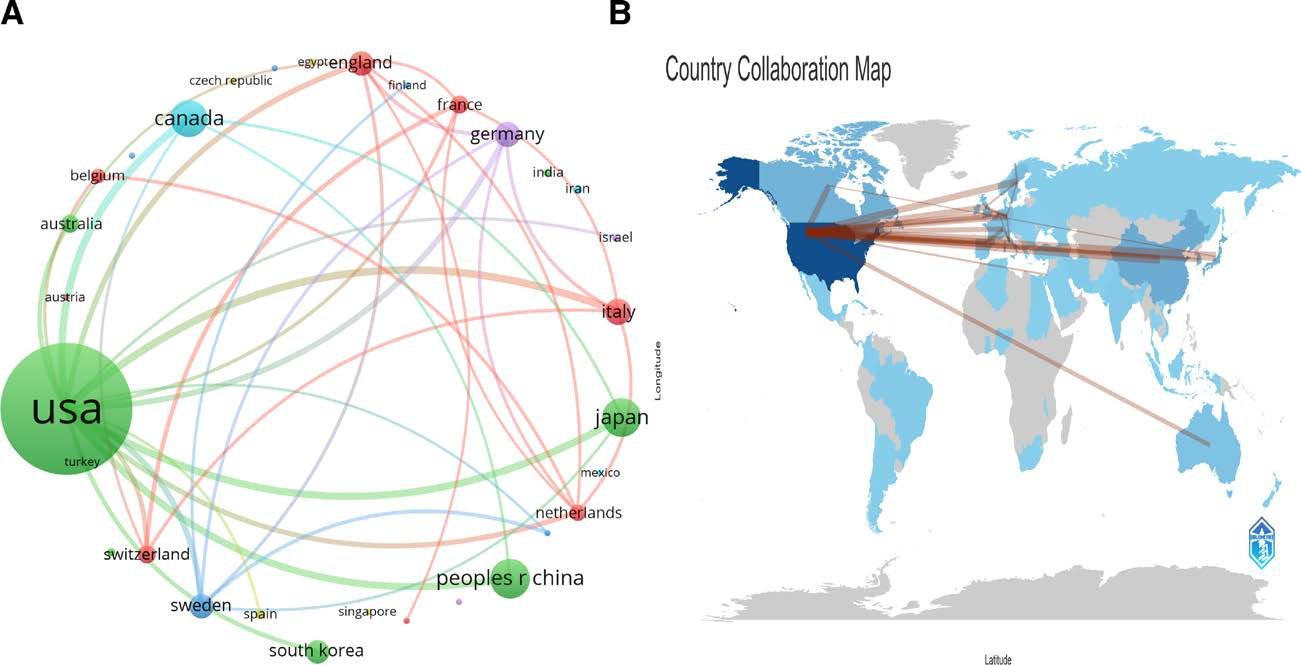
The visualization of countries (A) and geographical distribution (B) on IT.

The top 10 contributing countries and regions are identified through the analysis of cooperative network maps in IT-related research (Table 2 and Fig. 3). Leading the list with 3535 articles is the United States, followed by China (798), Japan (774), Canada (724), and Italy (484). The size of each circle in the network map represents the quantity of published papers for the respective nation, while the thickness of the connecting lines indicates the level of collaboration. This analysis reveals variations in the extent of IT research conducted across different parts of the world. Notably, the top 5 countries in terms of collaboration intensity include Sweden, Japan, Canada, Italy, and the United States. This suggests a growing trend of cross-border and international research collaboration in the field.

**Table 2:** The top 10 productive countries/region on the research of IT.

Ranking	Countries/Region	Publications	Link strength
1	USA	3535	151,343
2	China	798	14,303
3	Japan	774	21,531
4	Canada	724	38,976
5	Italy	484	22,629
6	Germany	461	18,223
7	Sweden	445	18,804
8	England	438	15,769
9	South Korea	426	9487
10	Switzerland	304	11,179

IT = islet transplantation.

**Figure 4.**
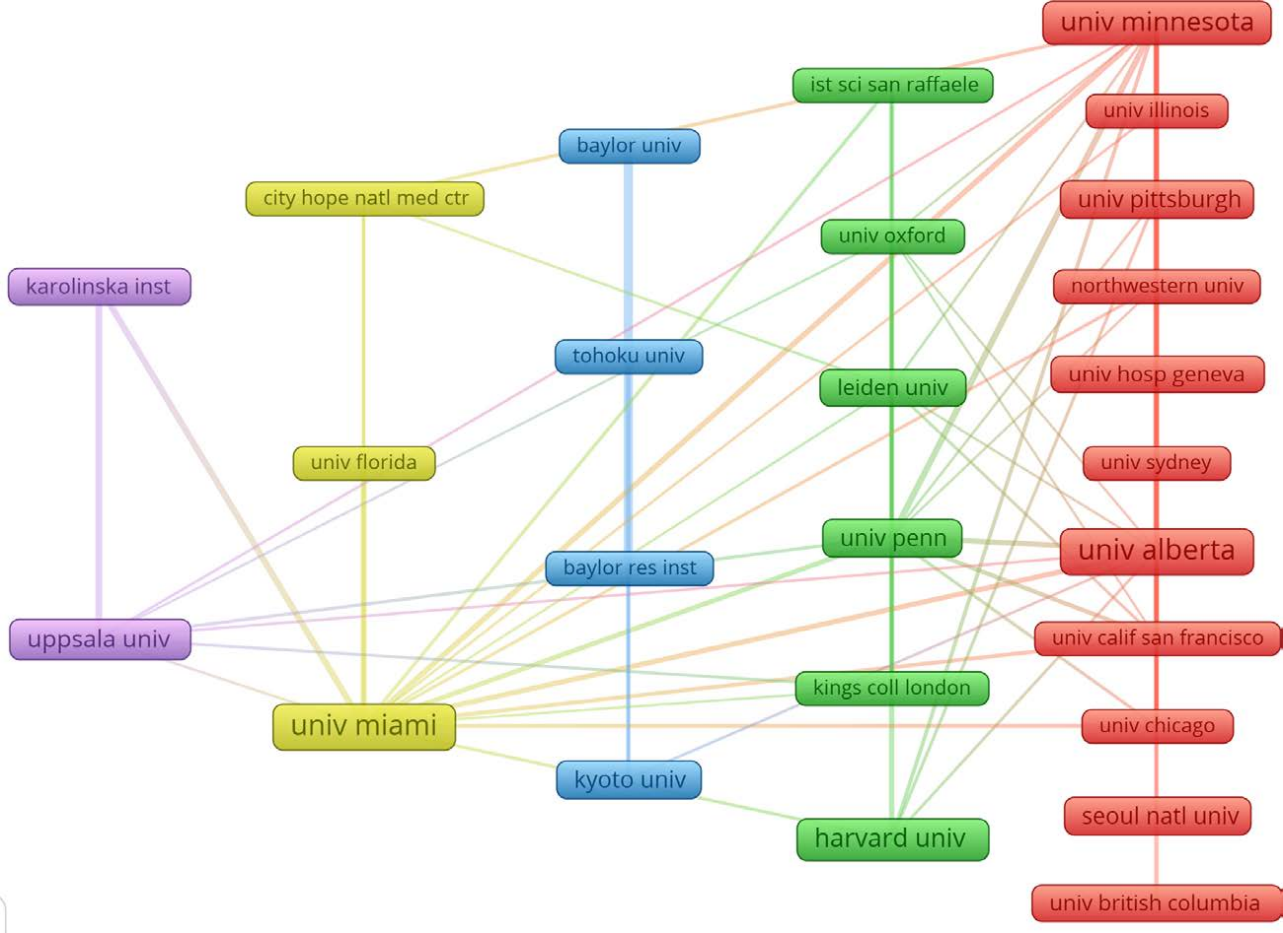
The network map of institutions.

As depicted in Table 3, the University of Alberta (378 articles), University of Miami (353 articles), and University of Minnesota (299 articles) rank as the top 3 universities actively supporting IT research. Following closely is Harvard University (251 articles). When examining the collaboration connection strength and the institutional network collaboration map (Fig. 4), it becomes evident that there is room for enhancing global cross-institutional collaboration in IT research.

**Table 3:** The top 10 productive institutions on the research of IT.

Ranking	Institutions	Publications	Link Strength
1	University of Alberta	378	380,456
2	University of Miami	353	313,029
3	University of Minnesota	299	241,551
4	Harvard University	251	195,647
5	Uppsala University	205	155,640
6	University of Pittsburgh	182	151,065
7	Kyoto University	176	153,218
8	Seoul National University	158	114,043
9	University of Pennsylvania	145	128,672
10	Karolinska Institute	134	98,889

IT = islet transplantation.

### 3.4. Analysis of leading authors and co-cited authors

**Figure 5.**
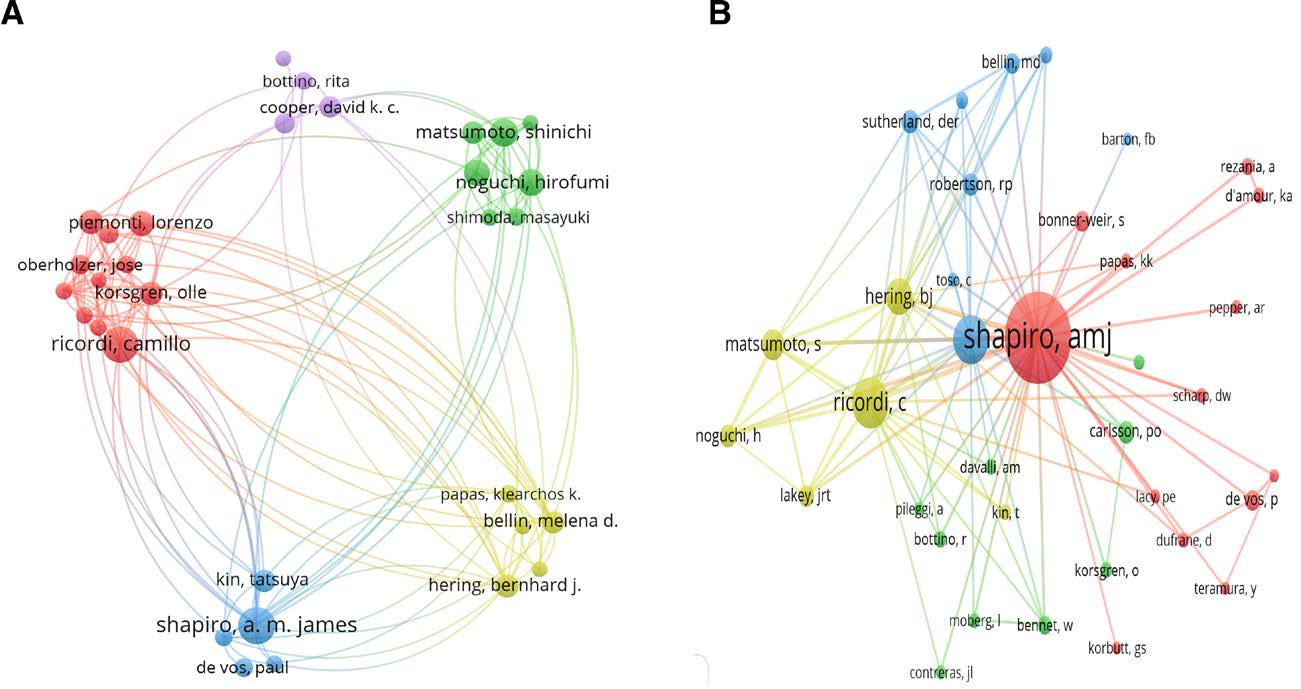
Cooperation map of authors.

In the author cooperation network’s co-occurrence map (Fig. 5A), distinct clusters are represented by different colored circles, and the lines indicate the intensity of collaboration. This analysis reveals the presence of 5 primary author collaboration groups, demonstrating substantial cooperation among authors. Table 4 highlights the top 5 authors in terms of the number of published documents, including Shapiro (126 articles), Ricordi (121 articles), Matsumoto (85 articles), Noguchi (80 articles), and Naziruddin (79 articles). Figure 5B illustrates co-cited authors, with the most frequently co-cited authors being Shapiro (4808 co-citations), Ricordi (2285 co-citations), Ryan (2219 co-citations), Hering (1556 co-citations), and Matsumoto (1275 co-citations). These authors hold significant academic influence in the field of IT.

**Table 4:** The top 10 productive and cited authors on the research of IT.

Ranking	Author	Documents	Co-cited author	Count
1	Shapiro	126	Shapiro	4808
2	Ricordi	121	Ricordi	2285
3	Matsumoto	85	Ryan	2219
4	Noguchi	80	Hering	1556
5	Naziruddin	79	Matsumoto	1275
6	Ricordi	74	Sutherland	893
7	Piemont	73	Robertson	877
8	Berney	71	Noguchi	876
9	Hering	67	Carlsson	843
10	Korsgren	67	Lakey	797

IT = islet transplantation.

### 3.5. Analysis of keywords and burst words

**Figure 6.**
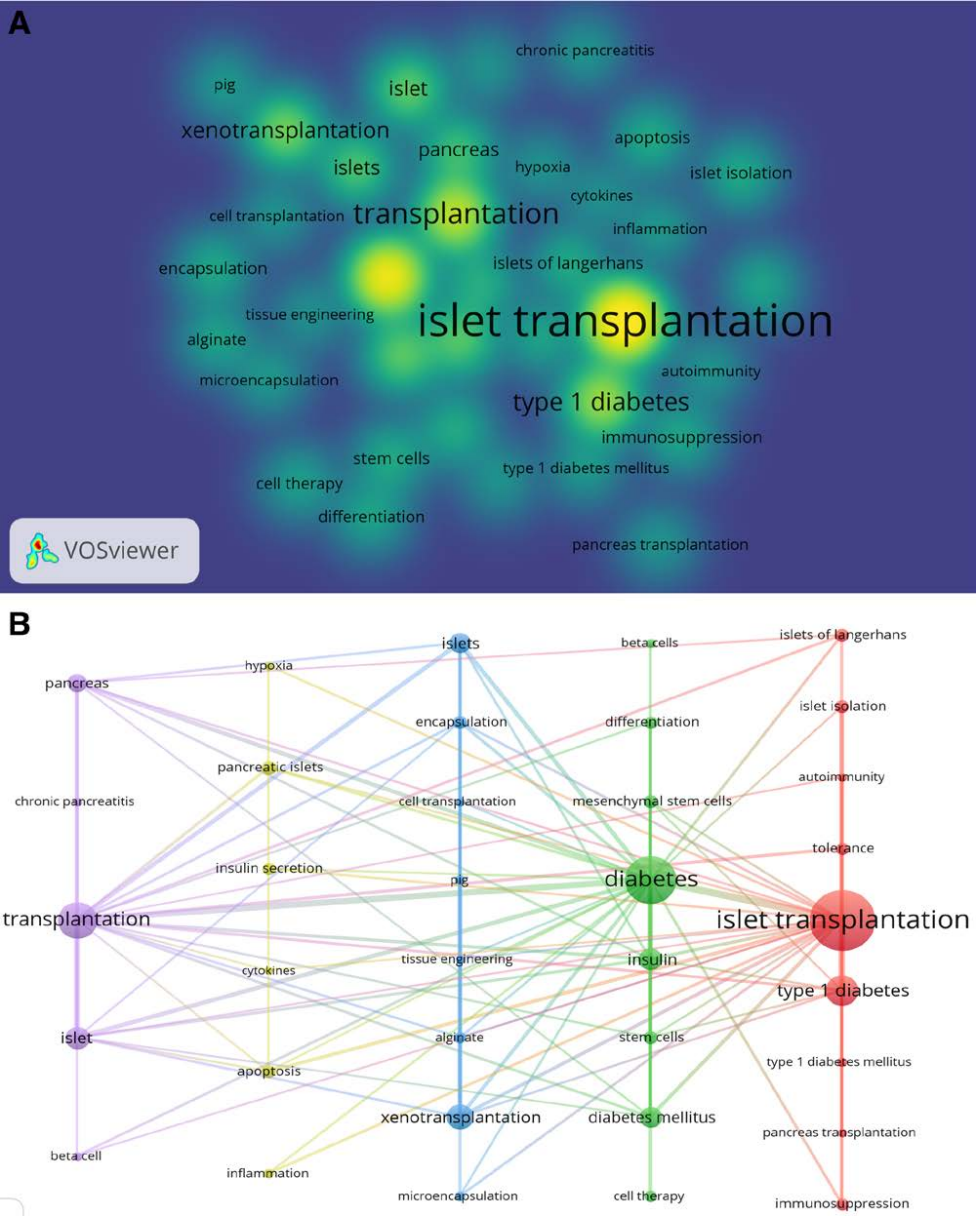
Analysis of keywords. (A) Keyword density map and (B) co-occurring map of keywords.

**Figure 7.**
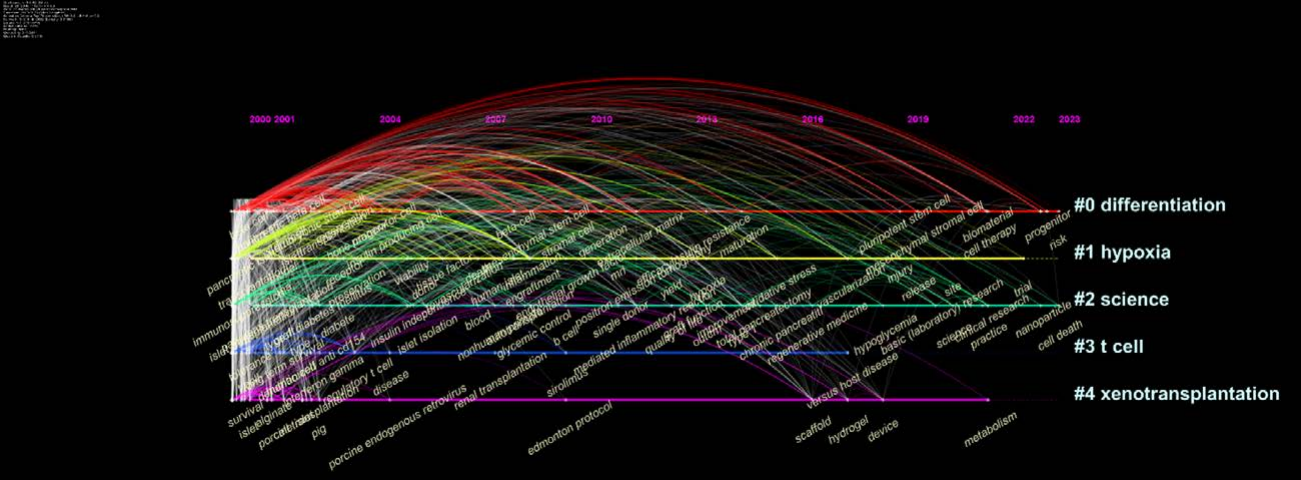
Keyword timeline chart and keyword clusters.

**Figure 8.**
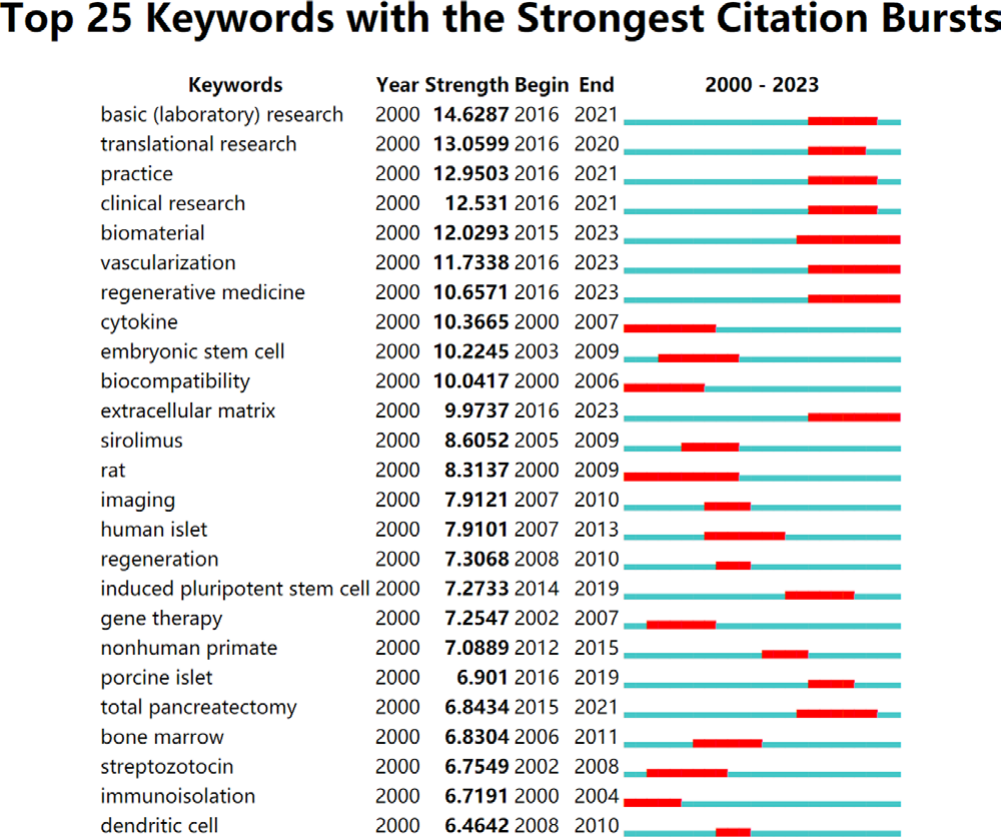
Top 25 keywords with the strongest citation bursts.

The key concepts within an article can be summarized and extracted through keywords, with increased frequency indicating a greater focus on the relevant subject of study. Therefore, by analyzing keywords, it is possible to identify research hotspots and shifts in emphasis.^[20]^ This study analyzed 112 keywords with a frequency of occurrence exceeding 30. Among the most frequently occurring terms were “IT” (1300 instances), “xenotransplantation” (383 occurrences), “apoptosis” (163 occurrences), “stem cells” (158 occurrences), “immunosuppression” (148 occurrences), and “microencapsulation” (105 occurrences). The research hotspots encompassed fundamental medicine, clinical medicine, and epidemiology, as illustrated in Figure 6A and B. To track research progress over time, the keywords were aggregated to create a timeline map (Fig. 7). The study resulted in the formation of 5 clusters: differentiation, hypoxia, science, T cells, and xenotransplantation. “Burst words” represent terms that experience a significant spike in frequency for a short period, indicating research hotspots. The emergence and disappearance timeframes of burst words provide insights into research trends and directions.^[21]^ In this study, Bursting Words focuses on the basic theory and clinical application of IT (Fig. 8).

### 3.6. Co-cited references and reference with citation bursts

**Figure 9.**
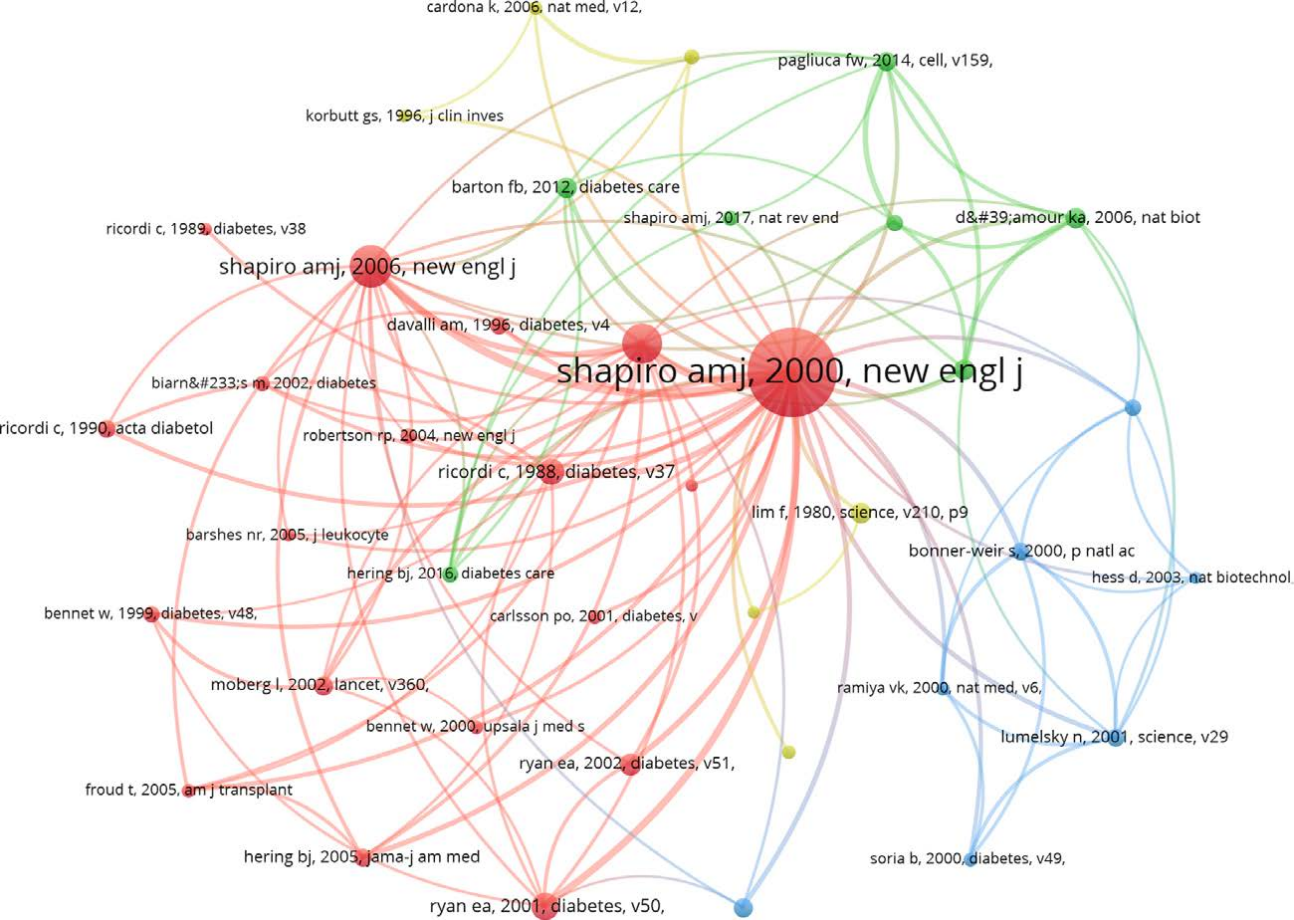
The visualization of co-cited references on research of IT. IT = islet transplantation.

Co-cited Citations: Over the past 30 years, there have been 154,892 co-cited references related to IT research. Each of the top 10 co-cited references listed in Table 5 has garnered a minimum of 141 co-citations, with one receiving more than 412 co-citations. The co-citation network map, composed of references with 150 or more co-citations, is depicted in Figure 8. Furthermore, Figure 9 highlights the active co-citations among references, including instances where “Shapiro, 2000, N Engl J Med” actively co-cites “Ryan, 2005, Diabetes,” “Ryan, 2001, Diabetes,” and “Ricordi, 1988, Diabetes,” among others.

**Table 5:** Top 10 co-cited references on research of IT.

Ranking	Co-cited reference	Citations
1	Shapiro AMJ, 2000, N Engl J Med, v343, p230	2748
2	Shapiro AMJ, 2006, N Engl J Med, v355, p1318	1023
3	Ryan EA, 2005, Diabetes, v54, p2060	942
4	Ryan EA, 2001, Diabetes, v50, p710	571
5	Ricordi C, 1988, Diabetes, v37, p413	551
6	Ryan EA, 2002, Diabetes, v51, p2148	435
7	Barton FB, 2012, Diabetes Care, v35, p1436	412
8	Kevin A D’Amour, 2006, Nat Biotechnol, v24, p1392	388
9	Kroon EJ, 2008, Nat Biotechnol, v26, p443	387
10	Lim FL, 1980, Science, v210, p908	384

IT = islet transplantation.

**Figure 10.**
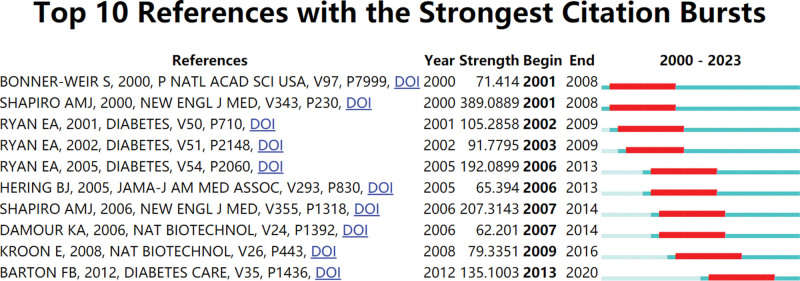
Top 10 references with strong citation bursts. A red bar indicates high citations in that year.

Citation burst references are those that have experienced a substantial surge in citations during a specific period, indicating heightened academic interest in a particular topic. In this study, CiteSpace identified 10 references with significant citation bursts, as illustrated in Figure 10. Each bar in Figure 10 corresponds to a year, with red bars highlighting periods characterized by a remarkable surge in citations.^[22]^ Citation bursts were observed spanning from as early as 2001 to as late as 2013. The reference “IT in Seven Patients with TD1 Mellitus Using a Glucocorticoid-Free Immunosuppressive Regimen” by Rajotte et al displayed the most substantial citation burst, with a strength of 389.08, occurring between 2001 and 2008. The reference “International Trial of the Edmonton Protocol for IT,” published in the New England Journal of Medicine by Shapiro et al, experienced notable citation bursts from 2004 to 2014, with a strength of 207.31, making it the second most impactful burst. These 10 references exhibit burst strengths ranging from 62.20 to 389.08 and have a duration of influence lasting between 6 and 7 years. For further details, please refer to Table 6, which outlines the primary research contents of these ten references, ordered in accordance with the literature in Figure 9.

**Table 6:** The main research contents of the 10 references with strong citations bursts.

Rank	Strength	Main research content
1	71.41	Human pancreatic duct tissue can be cultured and guided to develop in vitro into islet tissue capable of responding to glucose. This approach holds the potential to provide a new source of pancreatic islet cells for transplantation.^[23]^
2	389.08	The infusion of an adequate islet mass, along with glucocorticoid-free immunosuppression, can result in achieving insulin independence and maintaining excellent metabolic control.^[24]^
3	105.28	This approach is effective in managing labile diabetes and offers protection against undiagnosed hypoglycemia, particularly in carefully selected individuals.^[25]^
4	91.77	It presents a viable alternative for individuals experiencing severe hypoglycemia or glycemic instability.^[26]^
5	192.08	It can address issues related to hypoglycemia and glucose instability. The majority of participants maintained C-peptide secretion for up to five years, although many eventually required some insulin supplementation.^[27]^
6	65.39	Following single-donor, marginal-dose IT, this established transplant strategy effectively prevented hypoglycemia and restored insulin independence in all 8 recipients.^[28]^
7	207.31	In individuals with TD1 mellitus experiencing unstable control, IT using the Edmonton protocol can effectively restore long-term endogenous insulin production and promote glycemic stability.^[29]^
8	62.20	The insulin content of insulin-expressing cells generated from human embryonic stem cells closely resembles that of adult islets.^[30]^
9	79.33	Human embryonic stem cells have the capability to differentiate into insulin-secreting cells that can respond to glucose.^[31]^
10	135.10	Improvements in the major effectiveness and safety outcomes of IT between 1999 and 2006 for transplant patients vs recipients between 2007 and 2010.^[32]^

IT = islet transplantation.

## 4. Discussion

### 4.1. General information

With abnormally elevated blood glucose levels serving as an indicator of the endocrine disorder diabetes, it is projected that by 2045, approximately 693 million individuals worldwide will be affected by this condition,^[1]^ bearing a heavy financial burden.^[33]^ Many diabetic patients have discovered that IT is effective in reducing hyperglycemia and enabling them to achieve insulin independence. However, further research is needed to enhance its long-term success rate.^[34]^ As a result, IT has been a hot topic of research in diabetes treatment for more than 2 decades.

From 2000 to 2003, the annual average of published papers was <300, indicating that IT research was in its early stages with an insufficient foundational base. Subsequently, from 2004 to 2009, the field saw an increase in publications, averaging over 350 papers per year. However, from 2010 to 2013, there was a substantial surge in publications, with an annual average of 400 papers. In the past 5 years, the number of publications related to IT literature has remained relatively stable, suggesting that IT-related fields continue to be a prominent area of research.

The United States and China have emerged as leaders in IT research, with the United States publishing 3535 papers, followed by China with 798, and Japan with 774. Among the top 10 research organizations, the majority are based in the United States (*n* = 5, 50%). Significant cooperation is notably observed among the United States, Canada, Germany, and China, with Germany also actively collaborating with Italy, Japan, and Sweden. However, despite the existing collaborative relationships among certain nations, the extent and intensity of interinstitutional cooperation are not optimal. For instance, collaborations between institutions in Poland and China are limited, and if this continues, it may hinder the progress of the research field in the future. Therefore, we strongly advocate for research institutions worldwide to engage in broader cooperation and dialogue to collectively advance IT research.

“Transplantation” (Impact Factor = 6.20) is the primary publisher of IT research, establishing itself as the leading journal in this field of study. Among all the journals, “N Engl J Med” boasts the highest impact factor (Impact Factor = 158.50), followed by “JAMA” (Impact Factor = 120.70). A glance at co-cited publications reveals that many of them belong to high-impact Q1 journals, underscoring the pivotal role played by these prestigious global journals in fortifying IT research.

From an authorial standpoint, Shapiro, Ricordi, and Matsumoto stand out as prolific contributors. Shapiro has published 126 papers, including 5 that explored the role of stem cells in IT,^[35–39]^ one of these articles talks about the side effects and risks of islet transplants.^[40]^ Ricordi, author of 121 papers, has affirmed in 1 article that the greater omentum can be used as a site for IT.^[41]^ Matsumoto, with 85 published papers, confirms in 1 piece that improving allogeneic IT by suppressing T helper 17 cell and enhancing Treg with histone deacetylase inhibitors.^[42]^

Shapiro is the most frequently referenced co-cited author, with 4808 citations, followed by Ryan (2219 citations) and Ricordi (2285 citations). In a study by Shapiro et al (2003), it was found that pancreata obtained during a clinically relevant period of ischemia exhibit significantly increased islet yield, vitality, and endogenous glutathione levels when intraductal glutamine is injected. To enhance islet yields in clinical isolations, intraductal administration of glutamine at the moment of digestive enzyme delivery into the harvested pancreas may be a straightforward yet valuable technique.^[43]^ Ryan discovered in 2005 that proteinuria that develops during clinical IT may be resolved by increasing the dosage of tacrolimus and withdrawing sirolimus.^[44]^ Ricordi found in 2008 that even after islet graft failure, individuals with TD1 may regain consciousness of hypoglycemia because to the enhanced metabolic regulation brought about by IT.^[45]^

### 4.2. Hotspots and frontiers

Synthesized and summarized are research hotspots, developmental trajectories, timeline mapping, burst word mapping, and the frequency of keyword occurrences in the IT area.

#### 4.2.1. IT sites and procedures

The process of isolating pancreatic islets capable of secreting insulin, whether from autonomous sources or autologous donors, is a crucial step in pancreatic IT procedures. This isolation involves chemical processes such as collagenase and neutral protease digestion to release pancreatic islets from the pancreas.^[46,47]^ Subsequently, the islets undergo refinement through various centrifugation steps to separate them from the ductal and acinar tissues of the pancreas.^[48,49]^ Due to its low morbidity and ease of access, the liver and portal vein have been acknowledged as the preferred location for IT. This is evident from the fact that the majority of clinical IT procedures are conducted in this area.^[50]^ The liver, through the portal circulation, can provide oxygen to the transplanted islets until revascularization occurs. Additionally, this approach facilitates the delivery of insulin to the intestines and liver. The initial proof of concept for pancreatic IT into the liver of 7 T1D patients was reported by Shapiro et al^[6]^ The anterior chamber of the eye has garnered significant attention as a potential alternative location for IT due to its highly vascularized oxygen supply, immune-privileged nature, and accessibility. Intraocular insulin therapy has been shown to be more effective and immune-modulating than the liver transplantation site in the long-term improvement of hyperglycemia in mouse and nonhuman primate models of TD1.^[51,52]^ Bone marrow stands out as an alternative option for pancreatic IT due to its unique environment. Its characteristics, such as being extravascular, well-vascularized, and well-protected from external shocks, make it an ideal location for IT. The survival of islets in this context relies on the presence of widespread vascularization that is not directly in contact with blood. The limitations related to size and quantity can be addressed through multiple transplantations at various locations, leveraging the widespread dispersion of bone marrow and its accessibility. Another potentially less invasive treatment option is bone marrow transplantation, which carries low risk and provides easy access for bone aspiration biopsy samples.^[53]^

**Figure 11.**
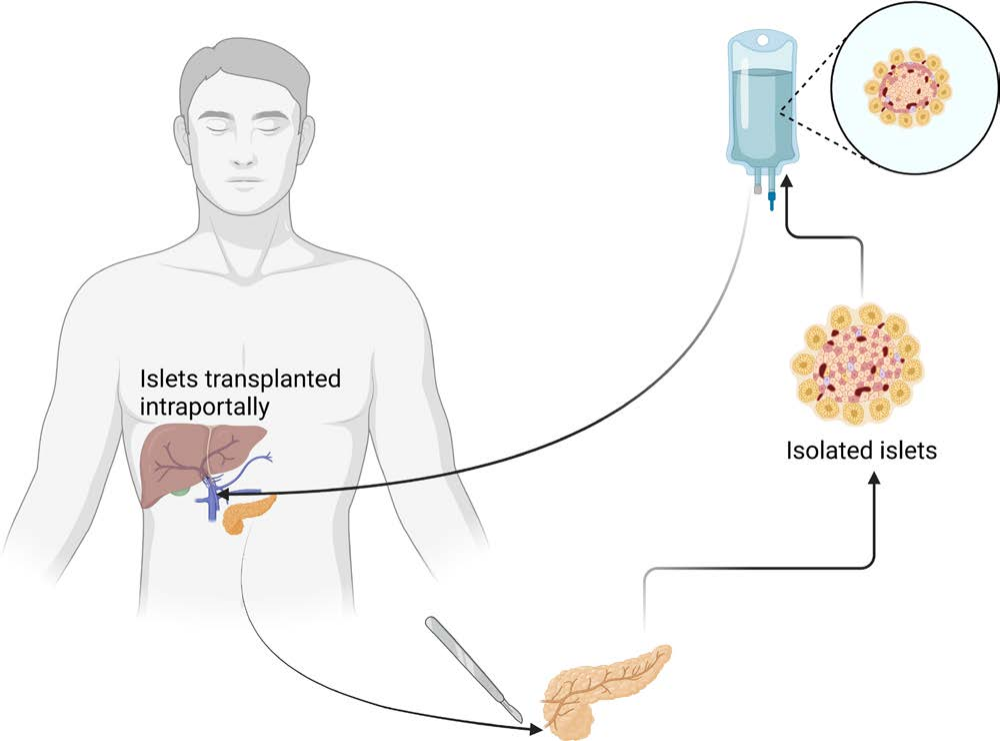
Procedure for IT. IT = islet transplantation.

The standard approach involves obtaining a pancreas from a deceased donor without diabetes. The acquisition, preservation, and transportation of deceased donor pancreases for IT require the same care and speed as pancreases for whole-organ transplantation.^[54]^ In total pancreatectomy with islet autograft, the diseased pancreas, along with the duodenum and spleen that share an arterial blood supply, is removed. After performing gastrojejunostomy and choledochojejunostomy, the pancreas is then transported to the Current Good Manufacturing Practice islet isolation facility. Here, acini are separated from endocrine cells through collagenase digestion and variable centrifugation purification to maximize the islet recovery rate while minimizing graft tissue volume. The final islet product is transplanted into the portal vein by reconnecting it to the stump of the splenic vein, the mesenteric vein, or the umbilical vein.^[55]^ The procedure for IT is shown in Figure 11.

#### 4.2.2. Novel therapies in IT

The acute loss of islets following transplantation has been attributed to poor islet vasculature and the hypoxic environment during the early posttransplantation phase, leading to ischemia.^[56]^ Hence, the search for novel and effective immunosuppressive therapies that can promote vascularization and inhibit the host immunological response is of utmost importance. In vitro studies have shown that mesenchymal stem cells (MSCs) serve as pleiotropic immune modulators through mechanisms such as direct cell–cell contact, mitochondrial transfer, creation of a cytoprotective environment, and the release of immunomodulatory molecules that influence various immune cell types, including T lymphocytes, B lymphocytes, natural killer cells, dendritic cells, and macrophages. These actions of MSCs contribute to the inhibition of ongoing immune processes and the enhancement of tissue engraftment, as well as the promotion of β cell viability.^[57]^ The trophic and angiogenic factors produced by MSCs hold the potential to alleviate early islet damage. Additionally, MSCs’ immunomodulatory properties, achieved through the release of cytokines that inhibit autoreactive T cells, can help reduce autoimmune responses.^[58]^ These attributes can be harnessed in in vivo co-transplantation strategies to support prolonged graft survival and facilitate islet engraftment. Multiple animal studies have demonstrated that co-transplanting MSCs with islets leads to improved outcomes in IT and the preservation of glucose homeostasis.^[59,60]^

The potential of microencapsulation is particularly noteworthy for its role in “stealth” transplantations, which can be customized for specific diseases by integrating advancements in cell therapies and nanomedicine for cell encapsulation.^[61]^ Increasing evidence suggests that encapsulated islets can survive, secrete insulin in vivo, and remain shielded from the host’s immune system. A pioneering clinical trial with encapsulated islets, conducted by Calafiore et al 2003, involved ten patients with TD1.^[62]^ Microencapsulated IT could enhance the success rate of IT in various ways. This includes long-term immune isolation through microencapsulation, modulation of peri-graft immune responses, enhancement of material biocompatibility, selection of optimal transplantation sites, and improvement of oxygen and nutrient supply to the graft.^[63]^

The “pancreas-on-a-chip” primarily focuses on the endocrine aspect of the pancreas within a microfluidic chip and can serve as a standardized, real-time platform for assessing islet potency and quality.^[64]^ This advanced technology permits the encapsulation and cultivation of human pancreatic cells on a miniaturized platform, commonly referred to as a “chip,” thereby closely mimicking the islets’ physiological milieu in vivo. This compact device incorporates an array of finely etched or molded microchannels, interconnected to facilitate blood flow. Within this environment, the islet cells possess the capability to discern alterations in nutrient concentrations, subsequently secreting the requisite hormones to sustain normoglycemia.^[65]^ To date, pancreas-on-a-chip microfluidic platforms have primarily demonstrated their potential for long-term islet culture, offering a standardized method to study islet function. Future advancements in microfluidic design, incorporating imaging-compatible biomaterials and biosensor technology, could provide an innovative tool for predicting outcomes of IT.^[66]^

#### 4.2.3. Comparison of IT and other forms of diabetes management

**Figure 12.**
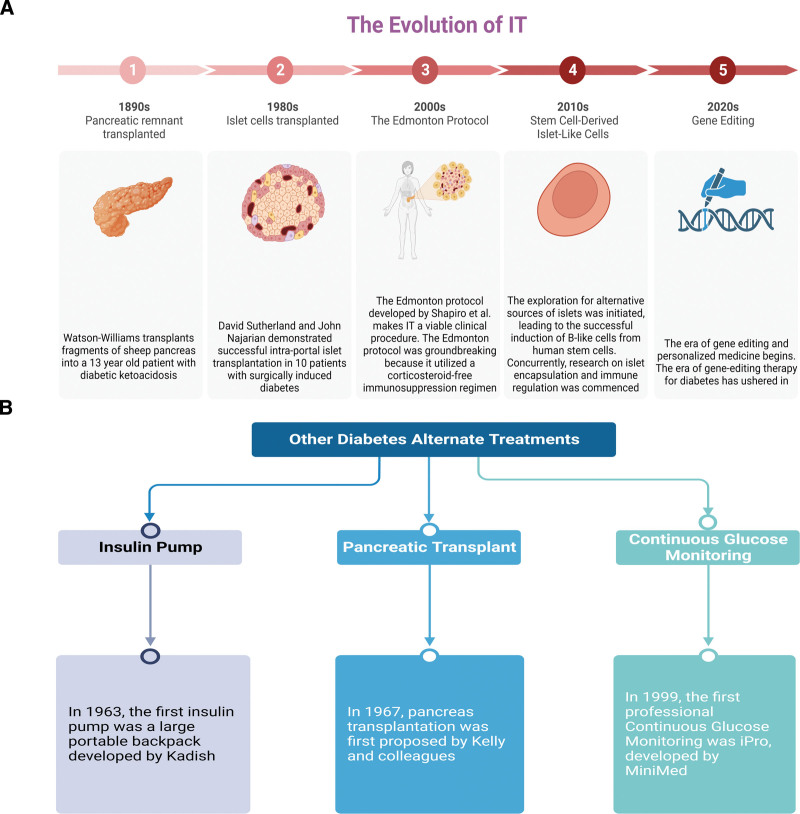
The evolution of diabetes treatment.

The treatment of diabetes encompasses various methods, including IT, continuous glucose monitoring, insulin pumps, and pancreas transplantation. The origins of IT can be traced to 1893 when Watson-Williams and Harsant conducted an experimental pancreas transplant from a sheep to a 13-year old in Bristol, UK. Tragically, the patient succumbed to acute ketoacidosis.^[67]^ Kadish introduced the initial version of an insulin pump in 1963. This bulky, backpack-like device was a sophisticated closed-loop system delivering insulin intravenously, regulated by ongoing blood glucose monitoring.^[68]^ A significant milestone was reached in 1980 by David Sutherland and John Najarian in Minnesota. They successfully performed intraportal IT in 10 patients with diabetes caused by surgical procedures.^[69]^ The idea of pancreas transplantation emerged in 1967, conceptualized by Kelly and his team.^[70]^ In 1999, MiniMed unveiled the first professional continuous glucose monitoring system, the iPro.^[71]^ The evolution of diabetes treatment is depicted in Figure 12, and the advantages and disadvantages of IT and other diabetes management methods are outlined in Table 7.

**Table 7:** Comparison of IT and other forms of diabetes management.

Methods of treatment	Advantages	Disadvantages	References
IT	1. Liberates patients from reliance on insulin. 2. Effectively mitigates hypoglycemia and stabilizes blood sugar levels. 3. Presents lower risks compared to whole pancreas transplantation in IT.	1. Prohibitive cost. 2. Requirement for continuous immunosuppressive treatment. 3. Insufficient donor availability to+ satisfy demand.	^[72,73]^
Insulin pump	1. Enhanced precision in insulin delivery. 2. Greater flexibility in insulin administration. 3. Improved glycemic regulation. 4. User-friendly design.	1. Increased costs. 2. Necessitates learning and adaptation. 3. Demands regular monitoring and adjustment.	^[74–76]^
Pancreas transplantation	1. Enables diabetic patients to regain normal blood glucose levels, eliminating the need for external insulin. 2. Substantially enhances blood pressure and lipid management. 3. Reverses microscopic lesions resulting from diabetes mellitus.	1. Multiple surgical complications. 2. Scarce pancreas transplant donor sources. 3. Increased surgical risk.	^[77,78]^
Continuous glucose monitoring	1. Enhanced understanding of blood glucose fluctuations and trends. 2. This may decrease both the frequency and duration of hypoglycemic episodes. 3. Helps in minimizing blood glucose volatility and stabilizing glucose levels. 4. Offers decision support for patients, aiding in better insulin dosage management and infusion.	1. Improved comprehension of blood glucose variability and patterns. 2. Potentially reduces the occurrence and length of hypoglycemic episodes. 3. Aids in reducing blood glucose fluctuation and enhancing stability. 4. Provides patient guidance, facilitating more effective insulin dosage and infusion adjustments.	^[79,80]^

IT = islet transplantation.

## 5. Strengths and limitations

This research is valuable as it offers a bibliometric analysis of the current state of IT research worldwide, offering scholars valuable insights into authors, journals, institutions, research focal points, and emerging frontiers. However, it is important to note that this study has limitations, as it exclusively relies on analyses from the WoSCC database, which is considered a highly reputable and reliable academic literature source. Additionally, the relatively limited number of articles included in the study is a result of the data collection method, which does not encompass meetings, books, and other types of publications.^[81]^

## 6. Conclusion

This bibliometric and visualized analysis, covering the period from January 1, 2000 to October 1, 2023, provides a comprehensive overview of the global scientific trends in IT for the treatment of diabetes mellitus. Our findings reveal a continuous increase in research output, with significant contributions from numerous countries, led predominantly by the United States and China. The areas identified as current research hotspots, including xenotransplantation, apoptosis, stem cells, immunosuppression, and microencapsulation, underscore the evolving landscape of IT research and its potential to address the complex challenges of diabetes treatment. Crucially, this study highlights the importance of evidence-based medicine in guiding the trajectory of IT research and practice. The analysis suggests a growing anticipation for advancements in IT procedures and novel therapies, reflecting a collective endeavor to enhance treatment efficacy and patient outcomes. However, it is evident that the field requires more rigorous, high-quality research, particularly randomized controlled trials and meta-analyses, to further substantiate the benefits and optimize the application of IT in clinical settings. The collaborative efforts illustrated in our research underscore the value of international cooperation in advancing the scientific and clinical frontiers of IT. Nonetheless, the path forward must prioritize the integration of evidence-based principles to ensure that IT not only remains at the forefront of diabetes research but also transitions into a viable, effective treatment option for patients worldwide. In conclusion, our study maps out the scientific landscape of IT over the past 2 decades, highlighting significant achievements and identifying critical areas for future research. Embracing evidence-based approaches will be pivotal in realizing the full potential of IT, offering hope for improved quality of life and outcomes for patients with diabetes mellitus. As the IT research community continues to explore innovative solutions, the emphasis on evidence-based medicine will ensure that these advancements are grounded in solid scientific rationale and clinical relevance, paving the way for the next generation of diabetes treatment.

## Author contributions

**Conceptualization:** Sheng Chen, Jianqiang Zhang.

**Data curation:** PeiZhong Wu, Ting Zhang.

**Formal analysis:** PeiZhong Wu, Hongjun Gao.

**Funding acquisition:** Hongjun Gao.

**Investigation:** Sheng Chen, Ting Zhang, Jianqiang Zhang.

**Methodology:** Jianqiang Zhang.

**Project administration:** Ting Zhang, Jianqiang Zhang.

**Resources:** Jianqiang Zhang.

**Software:** Sheng Chen, PeiZhong Wu.

**Supervision:** Jianqiang Zhang, Hongjun Gao.

**Visualization:** Sheng Chen.

**Validation:** PeiZhong Wu, Jianqiang Zhang.

**Writing – original draft:** Sheng Chen, Hongjun Gao.

**Writing – review & editing:** Ting Zhang, Hongjun Gao.
